# *Eucalyptus* essential oils inhibit the lipopolysaccharide-induced inflammatory response in RAW264.7 macrophages through reducing MAPK and NF-κB pathways

**DOI:** 10.1186/s12906-020-02999-0

**Published:** 2020-06-29

**Authors:** Chen-Lung Ho, Lan-Hui Li, Yueh-Chun Weng, Kuo-Feng Hua, Tz-Chuen Ju

**Affiliations:** 1 Division of Wood Cellulose, Taiwan Forestry Research Institute, Taipei, Taiwan; 2 Department of Laboratory Medicine, Linsen, Chinese Medicine and Kunming Branch, Taipei City Hospital, Taipei, Taiwan; 3 Department of Pathology, Tri-Service General Hospital, National Defense Medical Center, Taipei, Taiwan; 4 EMA program in College of Bioresources, National Ilan University, Ilan, Taiwan; 5 Department of Biotechnology and Animal Science, National Ilan University, Ilan, Taiwan; 6 Department of Medical Research, China Medical University Hospital, China Medical University, Taichung, Taiwan; 7 Department of Animal Science and Biotechnology, Tunghai University, No. 1727, Sec. 4, Taiwan Blvd., Xitun Dist, Taichung City, 40704 Taiwan

**Keywords:** Anti-inflammation, *Eucalyptus* essential oils, Macrophages, Cytokine, Lipopolysaccharide

## Abstract

**Background:**

*Eucalyptus* essential oils have been used in traditional medicine for centuries. It was reported that *Eucalyptus* leaves possess antioxidant and antimicrobial effects. Here, we investigated the anti-inflammatory activity of the essential oils extracted from the leaves of four different *Eucalyptus* species in RAW264.7 macrophages.

**Methods:**

Lipopolysaccharide (LPS)-activated RAW264.7 macrophages were used to evaluate the anti-inflammatory activity of the leaf essential oils of *Eucalyptus*. The cell survival was quantified by an Alamar Blue assay. Nitric oxide (NO) production was assessed by Griess reaction. TNF-α and IL-6 production were measured by enzyme-linked immunosorbent assay (ELISA). Nuclear factor-κB (NF-κB) transcriptional activity was measured by NF-κB reporter assay. Intracellular protein expression levels were determined by Western blot. The expression levels of inducible NO synthase (iNOS), cyclooxygenase-2 (COX-2), mitogen-activated protein kinase (MAPK), protein kinase C (PKC) and NF-κB pathway were measured by western blot in LPS-activated RAW 264.7 macrophage.

**Results:**

The essential oils extracted from *Eucalyptus citriodora* leaf exert the best NO inhibitory activity in LPS-activated RAW264.7 macrophages. The essential oils were fractionated into fractions A-H, and fraction F has been demonstrated to inhibit the expression levels of TNF-α, IL-6, NO, iNOS and COX-2 in LPS-activated RAW264.7 macrophages. Mechanistic analysis revealed that fraction F reduced the phosphorylation levels of ERK1/2, p38, PKC-α, PKC-ε and PKC-δ, and inhibited the NF-κB transcriptional activity. The chemical composition of Fraction F was determined by GC-MS.

**Conclusions:**

The discoveries made herein could help develop innovative nonsteroidal anti-inflammatory drugs with minimal side effects and strong efficacy. Clinical trials on these *Eucalyptus* leaf essential oils will help customize and optimize their therapeutic administration.

## Background

*Eucalyptus* trees are in the genus *Eucalyptus* of the *Myrtaceae*. There are > 700 *Eucalyptus* tree species worldwide [1]. They originated in Australia and Indonesia. The wood is used for pulp, fiber, fuel, furniture and construction. The essential oils are distilled from the leaves and used for medicinal purposes. *Eucalyptus* trees grow rapidly and adapt well to their surroundings. They were introduced into Taiwan in the 1980s primarily as sources of pulp for papermaking. *Eucalyptus* essential oils are widely used in traditional cold, fever and bronchitis remedies [2]. Vapor inhalation and oral ingestion of *Eucalyptus* essential oils treat suppurative and general respiratory tract infections such as asthma and chronic obstructive pulmonary disease (COPD) [3]. Previous research found that *Eucalyptus* essential oils inhibit *Trypanosoma* [4]. Lemon *Eucalyptus* essential oil has strong efficacy against *Amblyomma cajennense* and *Anocentor nitens* [5]. The essential oils in *Eucalyptus* leaves have antiseptic efficacy against *Escherichia coli* and *Staphylococcus aureus* [6]. Salem et al. proposed that essential oils from *E. citriodora* possesses strong bactericidal and fungicidal activities [7]. *Eucalyptus* essential oils are immunoregulatory, anti-inflammatory, antioxidant and analgesic. They also enhance the phagocytic capacity of monocytes and macrophages [3]. The essential oils from *E. camaldulensis* kill *Aedes aegypti* and *A. albopictus* larvae [1]. *Eucalyptus* essential oils neutralize free radicals and reactive oxygen species (ROS) [8]. Essential oils from *E. citriodora*, *E. globulus* and *E. tereticornis* also exert analgesic and anti-inflammatory activity [2].

Bacterial infections trigger immune responses in the human body and large amounts of proinflammatory substances are released. Moderate inflammatory reactions activate immune cells that kill pathogens and protect the body. However, excessive inflammatory reactions cause rapid cell death and lower blood pressure which results in ischemia, tissue necrosis, organ failure and death. Thus, controlling inflammatory reactions is a medical priority. Inflammatory reactions protect the body against pathogens but may also injure surrounding tissue [9]. Whereas mild inflammatory reactions are beneficial and protective, severe inflammation may be destructive as it induces high fever, delirium and sepsis. Early acute inflammatory reactions are generally resolved after healing. However, they may persist and become chronic if they are dysregulated. Chronic inflammation may cause and/or exacerbate atherosclerosis [10], diabetes [11], aging [12], neurodegeneration [13], and cancer [14].

Inflammation is therapeutically controlled mainly by steroidal and nonsteroidal anti-inflammatory drugs (NSAIDs). The former has numerous serious side effects and may induce and/or exacerbate hypertension, diabetes, glaucoma, hypertension, dermal atrophy, myopathy, hirsutism, insomnia, depression and mental illness. In contrast, the side effects of NSAIDs are relatively minor and may include gastrointestinal distress or inflammation. In severe cases, NSAIDs may cause GI perforation, peptic ulcers and hemorrhage. Current research has focused on the discovery of natural nonsteroidal anti-inflammatory fractions with minimal side effects and yet maximal efficacy. In the present study, an in vitro cell culture model was used to examine the influences of *Eucalyptus* essential oils on inflammatory reactions in macrophages. Other objectives were to identify the mutually interacting signal transduction pathways involved in these inflammatory reactions and determine whether *Eucalyptus* essential oils could mitigate the inflammatory response by modulating these biomolecular mechanisms.

*Eucalyptus* essential oils have been used in traditional medical treatments for centuries. They have been extensively studied and are valued for their comparatively low toxicity and broad-spectrum antiseptic activity [3, 6]. Here, we examined the anti-inflammatory activity of the essential oils extracted by water distillation from the leaves of *E. urophylla*, *E. grandis*, *E. camaldulensis* and *E. citriodora*. Lipopolysaccharides (LPS) were applied to RAW264.7 murine macrophages to trigger inflammatory reactions. We first screened the four types of *Eucalyptus* essential oils based on their inhibition of nitric oxide (NO) generation. The most active essential oil will be subjected to chromatographic analysis. Next, we tested the anti-inflammatory activity and elucidate the mechanisms by which they suppress the inflammatory response.

## Methods

### Plant materials

Fresh leaves of *Eucalyptus urophylla*, *Eucalyptus grandis* and *Eucalyptus camaldulensis* were collected in August 2017 from the Kukeng Experimental Plantation of the Taiwan Forestry Research Institute (TFRI) in south-central Taiwan (Yunlin County, elevation 100 m, N 23° 62′ 58˝, 120° 57′ 20˝), while fresh leaves of *Eucalyptus citriodora* were collected in August 2019 from the Lienhwachih Research Center, TFRI in central Taiwan (Nantou County, elevation 500 m, N 23° 91′ 75˝, 120° 88′ 52˝). The samples were compared with specimen No. E-0032, E-0033, E-0034 and E-0035 from the herbarium of National Chung Hsing University (NCHU) and were positively identified by Prof. Yen-Hsueh Tseng of NCHU. The voucher specimen (CLH-068, CLH-069, CLH-070 and CLH-071) were deposited in the NCHU herbarium. The collected leaves were immediately shipped to our Taipei headquarters, and the essential oils were extracted for subsequent analyses.

### Extraction of the leaf essential oils

A sample of 1 kg of fresh leaves from each species was placed in a round-bottomed flask, and 3 L of distilled water was added. After 8 h of steam distillation, the oil layers had separated from the water layers and were collected, and anhydrous sodium sulfate was added to eliminate the water. We repeated the isolation procedure for 4 times and combined the leaf essential oil for further fractionation. Yields of the essential oils were determined and the oils were stored in specimen bottles. Yields of leaf essential oils of *E. urophylla*, *E. grandis*, *E. camaldulensis* and *E. citriodora* were 3.18 ± 0.05, 2.98 ± 0.06, 3.58 ± 0.07, and 1.98 ± 0.08 ml/100 g, respectively.

### Fractionation of the leaf essential oil of *E. citriodora*

Fifty grams of *E. citriodora* leaf oil was mixed with 100 g silica gel (Merck 7734, Merck Co., Germany), separated on a silica gel open column (1000 g, 85 mm i.d., 850 mm length), and then eluted with a step gradient of n-hexane and ethyl acetate that ranged from n-hexane/ethyl acetate = 100:0–0:100. The fractions were collected by thin layer chromatography (TLC) and visualized at UV 254 nm and UV 366 nm, with iodine vapor and 1% vanillin/H_2_SO_4_. The band fractions from the leaf oil that contained the same compounds were combined to produce 8 sub-fractions: fractions A (5.3%), B (7.6%), C (11.5%), D (9.8%), E (10.2%), F (35.2%), G (18.1%), and H (2.3%).

### Gas chromatography-mass spectrometry (GC-MS) analysis of fractions F and G

A Hewlett-Packard HP 6890 gas chromatograph equipped with a DB-5 fused silica capillary column (30 m × 0.25 mm × 0.25 μm film thickness, J&W Scientific) and a FID detector were used for the quantitative determination of the chemical compositions of fractions F and G. Samples were incubated in an oven at 50 °C for 2 min, and then the temperature incrementally increased to 250 °C at 5 °C/min. The injection temperature was 270 °C. The carrier gas was helium and had a 1 ml/min flow rate. The detector temperature was 250 °C, with a split ratio of 1:10. The injection volume was 1 μl. Identification of the oil components was based on their retention indices and mass spectra obtained from GC-MS analysis on a Hewlett-Packard HP 6890/HP5973 equipped with a DB-5 fused silica capillary column (30 m × 0.25 mm × 0.25 μm film thickness, J&W Scientific). The MS reuslts were obtained in full scan mode with a 0.3 s scan time and a mass range of *m/z* 30–500 in the EI mode at 70 eV. All data are presented as the average of experiments performed in triplicate. Identification of the chemical compositions of fractions F and G was based on comparisons of Kovats index (KI), retention times (RT), and mass spectra with those obtained from authentic standards and/or the NIST and Wiley libraries spectra, and literature, respectively. The F and G fractions were identified using their Kováts retention indices calculated for all volatile constituents using a homologous series of n-alkanes C9-C23 on DB-5MS column, and comparing sample mass spectra with those obtained from authentic standards and/or the NIST and Wiley libraries spectra, and the literature [15].

### Materials

Antibodies against PKC-α, PKC-ε and PKC-δ, COX2, p-IKK-α and anti-p-IκB-α were procured from Santa Cruz Biotechnology (Dallas, TX). Antibodies against p-ERK1/2, p-JNK1/2 and p-p38 were purchased from Cell Signaling Technology (Danvers, MA). Antibodies against iNOS and β-actin, lysis buffer and polyvinylidene fluoride (PVDF) membrane were purchased from EMD Millipore (Bedford, MA). LPS (*Escherichia coli* O111:B4) and protease/phosphatase inhibitor cocktail were purchased from Sigma-Aldrich (St. Louis, MO). Enzyme-linked immunosorbent assay (ELISA) kits and enhanced chemiluminescence (ECL) reagents were purchased from Thermo Fisher Scientific (Waltham, MA). RPMI 1640 was purchased from Gibco Laboratories (Grand Island, NY, USA). Fetal bovine serum (FBS) was purchased from Biological Industries Ltd. (Kibbutz Beit Ha-emek, Israel). L-glutamine was purchased from Life Technologies (Carlsbad, CA, USA). Alamar Blue assay kit was purchased from AbD Serotec (Oxford, UK). QUANTI-Blue medium was purchased from InvivoGen (San Diego, CA).

### Cell cultures

RAW264.7 macrophage were purchased from the American Type Culture Collection (Rockville, MD). RAW-Blue macrophages were purchased from InvivoGen (San Diego, CA). All cells were propagated in RPMI 1640 supplemented with 10% (v/v) heat-inactivated FBS and 2 mM *L*-glutamine at 37 °C in a 5% CO_2_ incubator [16, 17].

### Cell viability assay

Cell death was quantified by the MTT reduction assay.

Cell viability was quantified by the Alamar Blue assay. Breifly, RAW264.7 macrophages were incubated with or without tested sample for 24 h. Alamar Blue reagent (10 μl) was added into the culture medium and incubated at 37 °C in a 5% CO_2_ incubator for 2–3 h. Fluorescence was detected with a fluorescence microplate reader (excitation/emission: 570 nm/600 nm; OPTImax tunable plate reader; Molecular Devices, Sunnyvale, CA, USA).

### NO production assay

RAW264.7 macrophages were incubated for 0.5 h with tested samples, followed by incubated with or without 1 μg/ml LPS for 24 h. NO levels in the culture medium were measured by the Griess reaction as described previously [17].

### Western blot analysis

Western blot was performed as described previously [16, 17]. Briefly, protein was extracted with a protease and phosphatase inhibitor cocktail containing lysis buffer. The lysates were electrophoretically fractionated through 10% SDS-polyacrylamide gel and then electrotransferred onto PVDF membranes. The membranes were incubated for 1 h at room temperature in 5% (v/v) nonfat milk in phosphate-buffered saline (PBS) with 0.1% (v/v) Tween-20). The primary antibodies were applied to the blots and incubated for 2 h at room temperature, followed by secondary antibody for 1 h at room temperature. The immunoreactive signals were detected with ECL reagents.

### NF-κB reporter assay

The NF-κB reporter assay was conducted as described previously [16, 17]. Briefly, RAW-Blue cells (10^5^ cells per 0.5 ml medium) were treated for 0.5 h with 12.5 μg/ml, 25 μg/ml, 50 μg/ml or 100 μg/ml Fraction F, followed by incubated with or without 1 μg/ml LPS for 24 h. The harvested medium (20 μl) was mixed with 200 μl QUANTI-Blue medium in 96-well plates and incubated at 37 °C for 2 h. SEAP activity was measured at 655 nm in an ELISA microplate reader (Bio-Tek Instruments, Winooski, VT, USA).

### ELISA assay

RAW264.7 macrophages (10^5^ cells per 0.5 ml medium) were treated for 0.5 h with tested sample, followed by incubation with or without 1 μg/ml LPS for 24 h. The levels of TNF-α and IL-6 in the culture medium were measured by ELISA according to the manufacturer’s protocol. Briefly, the 96-well microplates were coated overnight with anti-TNF-α or anti-IL-6 antibody, blocked with 1% (v/v) bovine serum albumin (BSA) in PBS, and washed repeatedly. Samples or standards (100 μl) were added to the microplates, incubated at room temperature for 2 h, washed repeatedly, incubated for 2 h with biotin-conjugated detection antibody, and incubated for 30 min with 100 μl streptavidin-horseradish peroxidase (HRP) plus substrate for signal development. Then 100 μl stop solution was added to each well and the OD_450_ were measured in an ELISA microplate reader (Bio-Tek Instruments, Winooski, VT, USA) [16, 17].

### Statistical analysis

Data were expressed as means ± standard deviation (SD) of triplicate samples. Each experiment was repeated at least three times to confirm reproducibility. Multiple groups were analyzed with one-way analysis of variance (ANOVA), followed by a post hoc Student-Newman-Keuls test. Differences among treatment means were considered statistically significant when *P* < 0.05.

## Result

### Effects of leaf essential oils from different Eucalyptus species on NO generation and cell viability

**Fig. 1:**
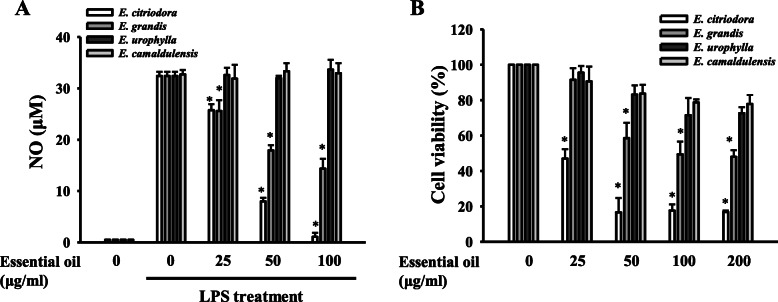
Effects of leaf essential oils from different *Eucalyptus* species on NO generation and cell viability. **a** RAW264.7 macrophages were incubated for 0.5 h with 25 μg/ml, 50 μg/ml, 100 μg/ml or 200 μg/ml *Eucalyptus* essential oils, followed by incubated with or without 1 μg/ml LPS for 24 h. NO levels in the culture medium were measured by the Griess reaction. **b** RAW264.7 macrophages were incubated with or without 25 μg/ml, 50 μg/ml, 100 μg/ml or 200 μg/ml leaf *Eucalyptus* essential oils for 24 h. Cell viability was determined by the Alamar Blue assay. Data are expressed as means ± SD for three independent experiments. * *P* < 0.05 compared to LPS alone. (*n* = 3)

NO shows a crucial role in LPS-induced inflammatory reactions in macrophages. Thus, we evaluated the effect of the leaf essential oils extracted from *E. citriodora, E. grandis, E. urophylla* and *E. camaldulensis* on NO production in LPS-activated macrophages. RAW264.7 macrophages were incubated with or without 25, 50 or 100 μg/ml essential oils for 0.5 h followed by LPS stimulation for 24 h (each group containing 0.1% DMSO in the cultural medium). We found that leaf essential oils from *E. grandis* and *E. citriodora* reduced the NO production in a dose dependent manner, while *E. citriodora* was potent than *E. grandis*. However, LPS-induced NO production was not affected by leaf essential oils from *E. urophylla* and *E. camaldulensis* (Fig. 1a). We next investigated the influences of four leaf essential oils on the cell viability by Alamar Blue assay. RAW264.7 macrophages were incubated with or without 25, 50 or 100 μg/ml essential oils for 24 h. We found that leaf essential oils from *E. citriodora* and *E. grandis* reduced RAW264.7 macrophages viability in a dose dependent manner; however, the viability was not significantly reduced by the leaf essential oils from *E. urophylla* and *E. camaldulensis* (Fig. 1b).

### Subfractions of *E. citriodora* leaf essential oils inhibited NO generation

**Fig. 2:**
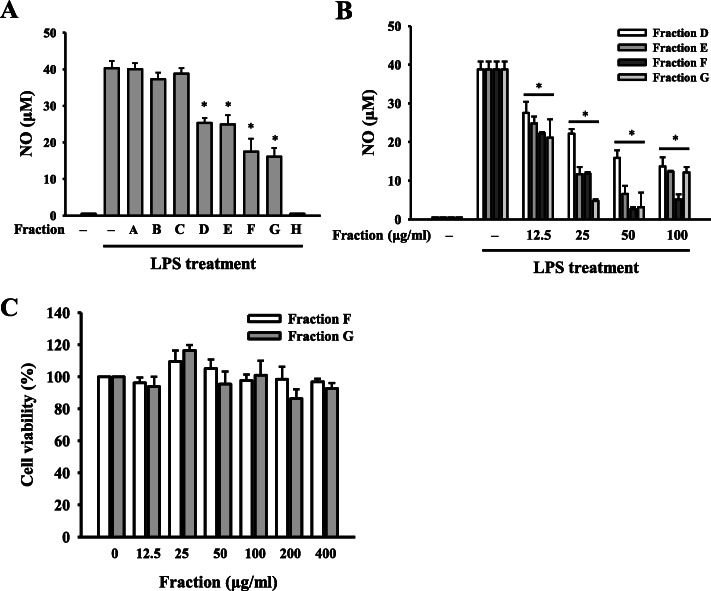
Subfractions of *E. citriodora* leaf essential oils inhibited NO generation. (**a**) RAW264.7 macrophages were treated for 0.5 h with 100 μg/ml Fractions A, B, C, D, E, F, G and H, followed by incubation with or without 1 μg/ml LPS for 24 h. NO production in the culture medium was measured by the Griess reaction. **b** RAW264.7 macrophages were treated for 0.5 h with 12.5 μg/ml, 25 μg/ml, 50 μg/ml or 100 μg/ml Fractions D, E, F or G, followed by incubation with or without 1 μg/ml LPS for 24 h. NO production in the culture medium was measured by the Griess reaction. **c** RAW264.7 macrophages were incubated for 24 h with or without Fractions F and G. Cell viability was measured by the Alamar Blue assay. Data are expressed as means ± SD of three independent experiments. *P < 0.05 compared to LPS alone. (*n* = 3)

As the leaf essential oils from *E. citriodora* had the most potent NO inhibitory activity in LPS-activated RAW264.7 macrophages, the essential oils were fractionated into Fractions A- H. We found that Fractions D, E, F, G and H significantly reduced NO generation to 63, 62, 42, 37 and 0.1%, respectively, in LPS-activated RAW264.7 macrophages (Fig. 2a). Although fraction H exerts the best NO inhibitory activity, the yield of the fraction H (2.3%) is too low to test its biological activity. Therefore, fractions D, E, F and G were selected for further investigation. The NO inhibitory activity of Fractions D, E, F and G were confirmed in a dose response study, and Fractions F and G exert better NO inhibitory activity than Fractions D and E (Fig. 2b). In addition, Fractions F and G did not affect the cell viability at the concentration below 400 μg/ml (Fig. 2c). These results indicated that Fractions F and G inhibited NO production was not due to the cytotoxicity. Furthermore, the chemical composition of Fractions F and G was analyzed by GC-MS, and the major compound in Fractions F and G was 4-methylsyringol (Table 1).

**Table 1:** Chemical composition of Fractions F and G

Compound ID	Kováts retention indices^a^	Kováts retention indices^b^	Concentration (%)		Identification^c^
			Fraction F	Fraction G	
Menthol	1171	1172	4.6	17.6	MS, KI, ST
4-hydroxy-Benzenemethanol	1337	1337	24.4	2.2	MS, KI, ST
Citronellic acid	1313	1311	12.7	2.4	MS, KI, ST
4-Methylsyringol	1449	1451	41.3	25.7	MS, KI, ST
*trans, cis*-Iridolactone	1446	1446	7.6	- ^d^	MS, KI
14-hydroxy-α-Humulene	1714	1714	–	17.5	MS, KI
Manool	2057	2057	1.8	0.8	MS, KI, ST
2-Phenyl ethyl anthranilate	2126	2127	2.2	0.9	MS, KI
Citronellyl anthranilate	2180	2181	1.1	0.5	MS, KI, ST
Geranyl anthranilate	2217	2215	1.1	0.6	MS, KI, ST

^a^ Kováts retention indices on a DB-5 column with reference to n-alkanes [15].

^b^ Kováts retention indices, experimental: n-alkanes (C9-C24) were used as reference points in the calculation of relative retention indices.

^c^ MS, NIST and Wiley library spectra and the literature; KI, Kovats index; ST, authentic standard compounds.

^d^ Not detected

### Fraction F inhibited proinflammatory mediators

**Fig. 3:**
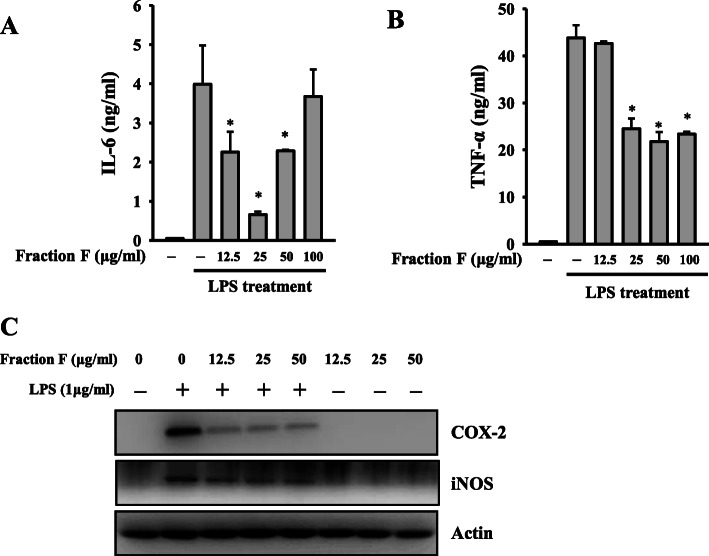
Fraction F inhibited proinflammatory mediators. RAW264.7 macrophages were treated for 0.5 h with 12.5 μg/ml 25 μg/ml, 50 μg/ml or 100 μg/ml Fraction F, followed by incubation with or without 1 μg/ml LPS for 24 h. The levels of IL-6 (**a**) and TNF-α (**b**) in the culture media were determined by ELISA. The expression levels of COX-2 and iNOS in the cell lysates were measured by Western blot (**c**). Western blot results are representative of three different experiments. ELISA data are presented as means ± SD of three independent experiments. *P < 0.05 compared to LPS alone. (*n* = 3)

Although Fractions F and G showed similar NO inhibitory activity, the yield of Fraction F (35.2%) was better than Fraction G (18.1%). Therefore, Fraction F was selected for further evaluation. We found that Fraction F at 12.5, 25 and 50 μg/ml reduced the expression levels of IL-6 to 55, 15 and 55%, respectively, in LPS-activated RAW264.7 macrophages (Fig. 3a). Fraction F at 12.5, 25, 50 and 100 μg/ml reduced the expression levels of TNF-α to 97, 55, 48 and 53%, respectively, in LPS-activated RAW264.7 macrophages (Fig. 3b). In addition, Fraction F also reduced the expression levels of COX-2 and iNOS in LPS-activated RAW264.7 macrophages (Fig. 3c).

### Fraction F reduced NF-κB activation

**Fig. 4:**
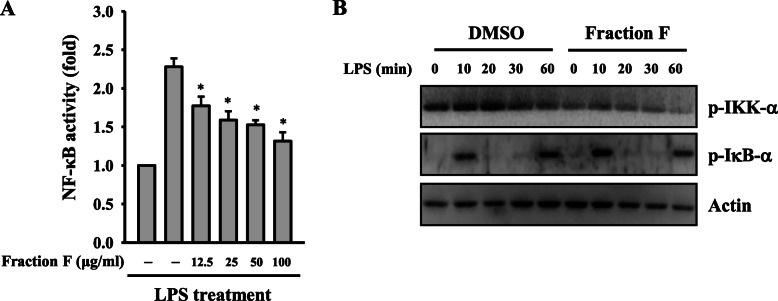
Fraction F reduced NF-κB activation. **a** RAW-Blue cells were treated for 0.5 h with 12.5 μg/ml, 25 μg/ml, 50 μg/ml or 100 μg/ml Fraction F, followed by incubated with or without 1 μg/ml LPS for 24 h. NF-κB transcriptional activity were measured by NF-κB reporter assay. **b** RAW264.7 macrophages were treated with 25 μg/ml Fraction F, followed by incubated with or without 1 μg/ml LPS for 0–60 min. The phosphorylation levels of IKK-α and I-κB-α in the cell lysates were assayed by Western blot. NF-κB transcriptional activity are expressed as means ± SD of three independent experiments. Western blot results are representative of three different experiments. *P < 0.05 compared to LPS alone

The transcription factor NF-κB plays a pivotal role in inflammation [18]. NF-κB inhibition suppresses NO generation and cytokine secretion in LPS-challenged macrophages [19]. Using NF-κB reporter cells (RAW-Blue macrophages), we found that NF-κB transcriptional activity was reduced to 78, 69, 65 and 55% by Fraction F at 12.5, 25, 50 and 100 μg/ml, respectively, in LPS-activated macrophages (Fig. 4a). To confirm the NF-κB inhibitory activity of Fraction F, the effect of Fraction F on the phosphorylation levels of IKK-α and IκB-α in LPS-activated RAW264.7 macrophages was investigated [20]. We found that Fraction F inhibited the phosphorylation levels of IKK-α and IκB-α in LPS-activated RAW264.7 macrophages (Fig. 4b).

### Fraction F reduced the phosphorylation levels of MAPK and PKC

**Fig. 5:**
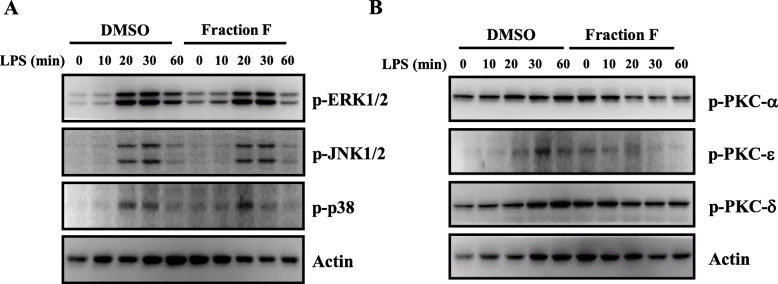
Fraction F reduced the phosphorylation levels of MAPK and PKC. RAW264.7 macrophages were treated with 25 μg/ml Fraction F, followed by incubated with or without 1 μg/ml LPS for 0–60 min. The phosphorylation levels of ERK1/2, JNK1/2 and p38 (**a**), and phosphorylation levels of PKC-α, PKC-ε and PKC-δ (**b**) in the cell lysates were assayed by Western blot

It has been demonstrated that LPS increased the expression levels of inflammatory mediators via the MAPK and PKC signaling pathways in macrophages [19]. We examined the effect of Fraction F on the phosphorylation levels of MAPK and PKC in LPS-activated macrophages. LPS increased the phosphorylation levels of ERK1/2, JNK1/2 and p38, and these effect were slightly reduced by Fraction F (Fig. 5a). In addition, LPS increased the phosphorylation levels of PKC-α, PKC-δ, and PKC-ε, and these effect were significantly reduced by Fraction F (Fig. 5b).

## Discussion

Inflammation is therapeutically controlled mainly by steroidal and nonsteroidal anti-inflammatory drugs. However, both cause several serious side effects. Current research has focused on the discovery of natural nonsteroidal anti-inflammatory drugs with strong therapeutic efficacy that induce minimal adverse reactions. In this study, of the four *Eucalyptus* species whose leaf essential oils we tested and compared, those from *E. citriodora* most effectively reduced the NO production induced by LPS in murine RAW264.7 macrophages. However, using a cell viability assay we found that the leaf essential oils of *E. citriodora* was toxic to the RAW264.7. To remove the toxic ingredients in the leaf essential oils of *E. citriodora*, the leaf essential oils were fractionated into Fractions A-H by a silica gel column. We found that Fractions F and G keep the anti-inflammatory activity and were not toxic to the Raw264.7 macrophages. When inflammation occurs, monocytes are attracted to chemokines, enter the lesion, and transformed into macrophages that undergo phagocytosis and release proinflammatory cytokines such as IL-6 and TNF-α. It has been demonstrated that LPS induces proinflammatory cytokines through ERK1/2, JNK1/2 and p38 in macrophages [21, 22]. In addition, LPS-mediated PKC activation induces the downstream signaling pathways such as MAPK [23, 24] or NF-κB [25], that regulate the expression of IL-1β, IL-6,and TNF-α in macrophages. Here, Fraction F inhibited the phosphorylation levels of ERK1/2, p38, PKC-α, PKC-ε and PKC-δ as well as suppressed NF-κB activation. However, Fraction F did not suppress JNK1/2 phosphorylation. These results suggest that Fraction F attenuated the inflammatory response in LPS-activated macrophages partially through inhibiting the activation of ERK1/2, p38, PKC-α, PKC-ε, and PKC-δ and NF-κB.

Notably, although Fraction F inhibited LPS-induced IL-6 secretion at 12.5 and 25 μg/ml; the IL-6 inhibitory activity was loss at higher concentration 50 and 100 μg/ml (Fig. 3a). The contradictory effect of Fraction F in IL-6 secretion may due to the complicated chemical composition. Chemical composition analysis showed that Fraction F contained 9 compounds: 4-methylsyringol (41.3%), 4-hydroxyl-Benzenemethanol (24.4%), Citronellic acid (12.7%), *trans, cis*-Iridolactone (7.6%), Menthol (4.6%), 2-Phenyl ethyl anthranilate (2.2%), Manool (1.8%), Citronellyl anthranilate (1.1%) and Citronellyl tiglate (1.1%) (Table 1). Although 4-methylsyringol, 4-hydroxyl-Benzenemethanol and Citronellic acid were the major compounds in Fraction F, there were no biological activity reported yet for these three compounds. Menthol is the fourth most abundant compound in Fraction F, and it has been reported to exert anti-inflammatory activity by suppressing the production of LTB_4_, PGE_2_ and IL-1β in LPS-activated human monocytes [26]. In addition, Shahid et al. demonstrated that the level of urinary menthol was significantly reduced in interstitial cystitis patients compared to healthy controls [27]. Menthol inhibited the expression levels of TNF-α, IL-6, IL-1β and CCL3 in LPS-activated RAW264.7 macrophages through reducing the activation of AKT and NF-κB [27]. These results suggest that Menthol (4.6% in fraction F) should be one of the bioactive compound in fraction F that response for the anti-inflammatory activity; however, we cannot rule out the possibility that the anti-inflammatory activity of fraction F was resulted from the synergistic effect between essential oil components.

The biological functions of *Eucalyptus* leaf essential oils have been extensively investigated, including antimicrobial, antiviral, antidiabetic, antioxidant, antitumor and anti-inflammatory activities [28]. It has been demonstrated that *E. citriodora* essential oil exerts the anti-inflammatory and analgesic effects on formol-induced edema and acetic acid-induced abdominal cramps in Wistar rats, and the major compound of the *E. citriodora* essential oil is citronellal [29]. Juergens et al. demonstrated that 1,8-cineole, isolated from *Eucalyptus* essential oil, inhibits the production of TNF-α, IL-1β, leukotriene B4 and thromboxane B2 in LPS-activated human blood monocytes [30]. In addition, *E. globulus* oil reduces LPS-induced chronic bronchitis and mucin hypersecretion in rats; however, the active compound was not identified yet [31].

Based on the cell viability assay, Fraction F was non-toxic to the RAW264.7 macrophages under the experimental condition (12.5–400 μg/ml). However, Manool, a minor compound in the Fraction F, has been reported to exhibit higher cytotoxic activity against human cervical adenocarcinoma HeLa cells (IC50 = 6.7 ± 1.1 μg/ml) and human glioblastoma U343 cells (IC50 = 6.7 ± 1.2 μg/ml) than for the normal Chinese hamster lung fibroblasts V79 cells (IC50 = 49.3 ± 3.3 μg/ml) [32]. Although Manool in Fraction F only 1.8%, the possible toxic effect of Fraction F should be further investigation. In addition, the anti-inflammatory activity of Fraction F in vivo, and the optimization of the administration routes, dosages and application intervals of Fraction F in animal study need further investigation.

## Conclusions

**Fig. 6:**
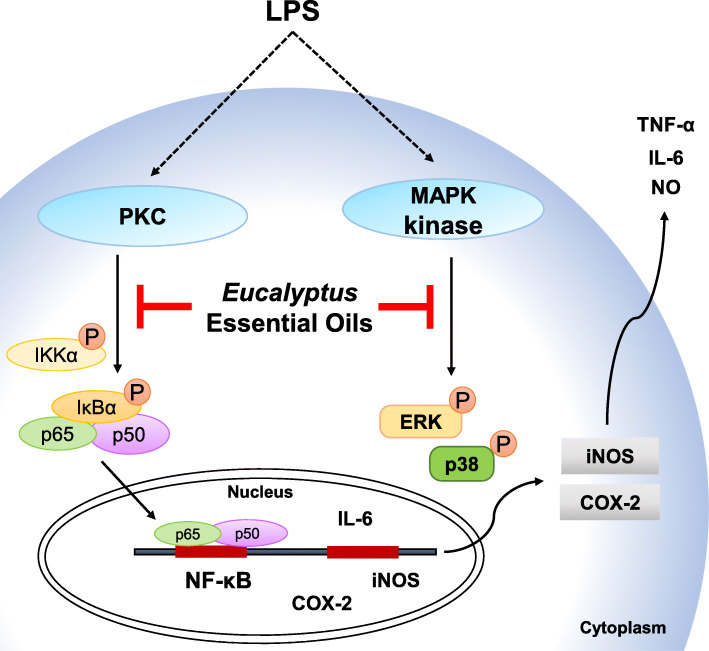
Overview of the putative mechanisms by which *E. citriodora* leaf essential oils attenuated the inflammatory response in macrophage. *E. citriodora* leaf essential oils inhibit LPS-induced inflammatory response in RAW264.7 macrophages by reducing the ERK1/2/p38 and PKC/NF-κB signaling pathways

Essential oils extracted from *Eucalyptus* leaves have antioxidant, antimicrobial, immunoregulatory, analgesic and anti-inflammatory properties. While conventional NSAIDs cause fewer serious side effects than their steroidal counterparts, they may nonetheless induce GI bleeding, distress, inflammation and ulceration in many patients, thereby reducing therapeutic compliance and treatment efficacy [33–35]. Here, we investigated the anti-inflammatory activity of the leaf essential oils from four *Eucalyptus* tree species in LPS-activated RAW264.7 macrophage. We demonstrated that the leaf essential oils from *E. citriodora* exert best NO inhibitory activity than other species. We further demonstrated that the Fraction F of *E. citriodora* leaf essential oils inhibited the expression levels of TNF-α, IL-6, NO, iNOS and COX-2 through reducing PKC/NF-κB and ERK1/2/p38 in the LPS-activated RAW264.7 macrophages (Fig. 6). These results suggested that leaf essential oils from *E. citriodora* can be developed as a anti-inflammatory agent in the future.

## Data Availability

All data of this study is included in the manuscript.

## Abbreviations

ANOVA: Analysis of variance
BSA: Bovine serum albumin
CC: Column chromatography
COPD: Chronic obstructive pulmonary disease
COX2: Cyclooxygenase II
ECL: Enhanced chemiluminescence
ELISA: Enzyme-linked immunosorbent assay
ERK: Extracellular signal-related kinase
FBS: Fetal bovine serum
FCS: Fetal calf serum
FID: Flame ionization detector
GC: Gas chromatography
IKK: IκB kinase
IL: Interleukin
iNOS: Inducible nitric oxide synthase
JNK: c-Jun *N*-terminal kinase
LPS: Lipopolysaccharides
MAPK: Mitogen-activated protein kinase
MS: Mass spectrometry
NF-κB: Nuclear factor kappa-light-chain-enhancer of activated B cells
NIST: National Institutes of Science and Technology
NO: Nitric oxide
p38: p38 mitogen-activated protein kinase
PBS: Phosphate-buffered saline
PKC: Protein kinase C
PVDF: Polyvinylidene fluoride
RIPA: Radioimmunoprecipitation assay
ROS: Reactive oxygen species
RPMI: Roswell Park Memorial Institute
SD: Standard deviation
SDS-PAGE: Sodium dodecyl sulfate-polyacrylamide gel electrophoresis
SEAP: Secreted embryonic alkaline phosphatase
TLC: Thin-layer chromatography
TNF: Tumor necrosis factor
WB: Western blot
